# Atypical presentation of renal cell carcinoma: a case report

**DOI:** 10.1186/1752-1947-1-26

**Published:** 2007-06-06

**Authors:** Deepak Doshi, Michael Saab, Nidhi Singh

**Affiliations:** 1 Emergency Medicine, Central Manchester and Manchester Children's University Hospitals, Oxford Road, Manchester M13 9WL, UK; 2 Emergency Medicine, Fairfield General Hospital, Rochdale Old Road, Bury, BL9 7TD, UK; 3 General Medicine, University Hospitals Birmingham, Raddlebarn Road, Birmingham, B29 6JD, UK

## Abstract

A case of Renal Cell Carcinoma (RCC) presenting to the Emergency Department with pyrexia and rigors is discussed.

## Case presentation

A 61 year old female patient presented to the Emergency Department with feeling unwell, pyrexia, nausea and headache. She gave a history of fever for the past three weeks with three episodes of rigors. She also gave a history of recent loss of appetite and some weight loss. She had no urinary problems.

On examination she looked pale. A mass was palpable in the right lower quadrant and lumbar region. Liver and spleen were not palpable. There were no signs of peritonitis.

Her pulse rate was 125/minute, BP 120/85 and temp 39.6 degree Centigrade. She had blood tests in the Emergency Department which revealed the following : Hb 12.0 g/dL, WCC 11.6 × 10(9)/litre and Platelets 237 × 10(9)/litre. Electrolytes and urea were as follows: Na 133 mmol/litre, K+ 4.2 mmol/litre, Urea 5.1 mmol/litre and Creatinine 78 micromol/litre. Chest X ray did not reveal any abnormality.

**Figure 1:**
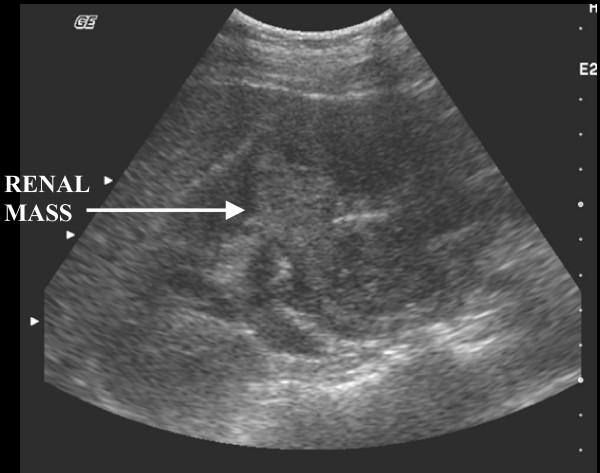
This is a picture of renal ultrasound. It shows echo-dense area in the renal area, suggestive of a renal tumour.

**Figure 2:**
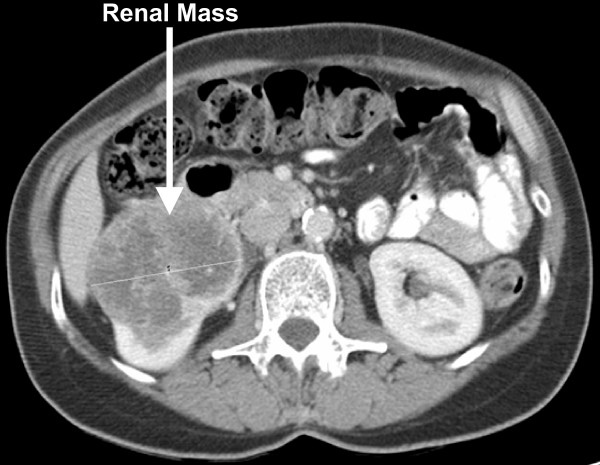
Computerised tomography scan image of abdomen showing renal mass.

The patient had Ultrasound Scan which showed mass in the right Kidney (Figure 1). Her subsequent CT scan as shown (figure 2) revealed a mass in her right kidney.

She underwent right sided nephrectomy after full staging procedures and appropriate investigations.

## Discussion

Malignant neoplasms involving the kidney may be primary or secondary tumors. Although metastatic lesions outnumber primary tumors, secondary renal neoplasms are usually clinically insignificant and are principally discovered at postmortem examination.

Patients with Renal cell carcinoma (RCC) present with a range of symptoms, but many are asymptomatic until the disease is advanced. At presentation, approximately 25 percent of individuals either have distant metastases or significant local-regional disease. Other patients, even some with only localized disease, present with a wide array of symptoms and/or laboratory abnormalities. Because of this unusual characteristic, RCC has been labelled the "internist's tumor"[1]. Today, most tumors are diagnosed incidentally [5,6].

The classic triad of flank pain, hematuria and flank mass is uncommon (10% cases) and is indicative of advanced disease. The frequency with which Renal Cell Carcinoma clinically presents is shown in table 1[2]. The literature has described several unique clinical presentations of Renal Cell Carcinoma in the form of hoarseness or calverial metastasis of which only five cases have been reported [3,4].

**Table 1:** Clinical presentation of Renal Cell Carcinoma

Haematuria	40%
Flank pain	40%
Palpable mass	25%
Classic Triad	10%
Weight loss	33%
Fever	20%
Hypertension	20%
Hypercalcemia	5%
Metastasis	30%

Renal cell carcinoma represents a heterogenous group of tumors, the most common of which is clear cell adenocarcinoma. RCC accounts for 3% of adult tumors. The incidence has increased more than 30% over the past two decades. It is generally accepted that the increased incidence rates reflect earlier diagnosis at an earlier stage, largely due to more liberal use of radiological imaging techniques. However advanced disease has also been diagnosed more frequently and mortality has increased as well [5].

Symptomatic presentation correlates with aggressive histology and advanced disease. Incidental tumours may be frequently detected in female and elderly patients, as these groups traditionally seek general medical care more regularly. The mode of presentation can independently predict an adverse patient outcome. Indicators of symptomatic presentations include flank pain, flank mass, varicocele, constitutional symptoms, paraneoplastic syndromes and bone pain related to metastatic disease [7].

Ultrasound scan was found to be useful screening test, but CT is the imaging study of choice to identify malignant features. MRI can be used in equivocal cases [7].

Pre-operative clinical variables may be used instead of the pathologic stage to determine the risk of recurrence [8].

## Conclusion

Renal cell carcinoma presents with various clinical features. Atypical presentations of RCC should be considered for patients presenting with pyrexia of unknown origin.

## Competing interests

The author(s) declare that they have no competing interests.

## Authors' contributions

DD wrote the first draft of the manuscript and scanned photographs for submission. MS proofread the case report and obtained patient consent. NS performed the literature search.
